# Analysis of the Skin Transcriptome in Two Oujiang Color Varieties of Common Carp

**DOI:** 10.1371/journal.pone.0090074

**Published:** 2014-03-06

**Authors:** Chenghui Wang, Michael Wachholtz, Jun Wang, Xiaolin Liao, Guoqing Lu

**Affiliations:** 1 Key Laboratory of Freshwater Fisheries Germplasm Resources, Ministry of Agriculture, Shanghai Ocean University, Shanghai, China; 2 Department of Biology, University of Nebraska at Omaha, Omaha, Nebraska, United States of America; 3 Key Laboratory of Ecological Impacts of Hydraulic-Projects and Restoration of Aquatic Ecosystem, Institute of Hydroecology, Ministry of Water Resources & Chinese Academy of Sciences, Wuhan, China; Temasek Life Sciences Laboratory, Singapore

## Abstract

**Background:**

Body color and coloration patterns are important phenotypic traits to maintain survival and reproduction activities. The Oujiang color varieties of common carp (*Cyprinus carpio* var. *color*), with a narrow distribution in Zhejiang Province of China and a history of aquaculture for over 1,200 years, consistently exhibit a variety of body color patterns. The molecular mechanism underlying diverse color patterns in these variants is unknown. To the practical end, it is essential to develop molecular markers that can distinguish different phenotypes and assist selective breeding.

**Methodology/Principal Findings:**

In this exploratory study, we conducted Roche 454 transcriptome sequencing of two pooled skin tissue samples of Oujiang common carp, which correspond to distinct color patterns, red with big black spots (RB) and whole white (WW), and a total of 737,525 sequence reads were generated. The reads obtained in this study were co-assembled jointly with common carp Roche 454 sequencing reads downloaded from NCBI SRA database, resulting in 43,923 isotigs and 546,676 singletons. Over 31 thousand (31,445; 71.6%) isotigs were found with significant BLAST matches (E<1e-10) to the nr protein database, which corresponds to 12,597 annotated zebrafish genes. A total of 70,947 isotigs and singletons (transcripts) were annotated with Gene Ontology, and 60,221 transcripts were found with corresponding EC numbers. Out of 145 zebrafish pigmentation genes, orthologs for 117 were recovered in Oujiang color carp transcriptome, including 18 found only among singletons. Our transcriptome analysis revealed over 52,902 SNPs in Oujiang common carp, and identified 63 SNP markers that are putatively unique either for RB or WW.

**Conclusions:**

The transcriptome of Oujiang color varieties of common carp obtained through this study, along with the pigmentation genes recovered and the color pattern-specific molecular markers developed, will facilitate future research on the molecular mechanism of color patterns and promote aquaculture of Oujiang color varieties of common carp through molecular marker assisted-selective breeding.

## Introduction

Body color and coloration patterns are important phenotypic traits associated with the survival and reproduction activities in many organisms, including their camouflage, mimicry, social communication and selective mating [1], [2], [3]. Coloration is determined mainly by pigments synthesized by chromatophores, i.e., pigment cells. So far, six types of pigment cells, including melanocytes (black, dark brown), xanthophores (yellow), erythrophores (red/orange), iridophores (reflecting), leucophores (white), and cyanophores (blue), have been reported in vertebrates [4]. Mammals and birds were found to have only melanocytes in their skin, whereas reptiles also possess xanthophores, erythrophores and iridophores. Interestingly, only teleost fishes were found to have all six types of pigment cells [5], [6], [7].

The molecular mechanism of melanin biosynthesis has been xextensively studied due to its biomedical significance; the melanogenesis pathways have been found conserved in vertebrates [8], [9], [10]. Melanin synthesis takes place within the melanosomes of melanocytes and involves the tyrosine metabolism pathway. The tyrosinases (TYR), tyrosinase-related protein 1 (TYRP1) and dopachrome tautomerase (DCT) are important enzymes in melanin synthesis [8], [11]. The most important regulator of melanogenesis is the MC1 receptor (MC1R) with its melanocortic peptides [10]. A single mutation of this gene was found to be associated with the beach mouse color pattern [12]. MC1R activates the cyclic AMP (cAMP) response-element binding protein (CREB), an important cellular transcription factor. Increased expression of microphthalmia-associated transcription factor (MITF) and its activation by phosphorylation (P) stimulate the transcription of TYR, TYRP1 and DCT [7], [8], [13], [14]. The biosynthesis of other types of pigments may involve different pathways and regulatory networks, but are in general less studied [6].

The common carp (*Cyprinus carpio* L.), a cyprinid belonging to Cyprinidae of ray-finned fishes (teleosts), is considered the most widely distributed and important aquaculture species in the world [15]. During the long history of domestication, numerous strains or variants of common carp have been established through artificial selection from the wild ancestral populations, of which many have diverse colors and coloration patterns [16], [17]. The molecular mechanisms underlying these diverse color patterns in common carp remain less well-understood.

Oujiang color varieties of common carp (*Cyprinus carpio* var. *color*), a unique variant of common carp, has been cultured in paddies and backyard ponds for about 1,200 years in the Oujiang river basin of Zhejiang province in China [18]. It has been found that several body colors and coloration patterns consistently exist, including whole red (WR), red with scattered black spots (RB), whole white (WW), and white with scattered black spots (WB), which provide an excellent model to explore the molecular mechanisms of pigment formation and development [16].

In a mating experiment involving whole red (WR) and whole white (WW) Oujiang color common carp varieties, we observed a ratio of three red to one white, providing evidence of Mendelian inheritance in the red and white body colors [19]. Although the black color showed dominant inheritance over red and white, it does not seem to follow the Mendelian law, suggesting that the regulation of this pattern is more complicated than the others (Wang et al., unpublished). In a study of red and black color inheritance with Koi, an ornamental variety developed from the common carp, three phenotypes were found associated with the red color and two phenotypes with the black one [20].

In our previous study, significant genetic differences were revealed among the different varieties of Oujiang color common carp through the use of microsatellite markers [21]. In addition, our field growth experiment demonstrated that RB grew faster than other body color types of Oujiang color common carp, while WW grew at the slowest rate [22]. Developing more genetic markers will not only facilitate the studies of population genetics of Oujiang color varieties of common carp, but it will also improve its aquaculture production through the identification of coloration patterns at early developmental stages.

In recent years, the transcriptome analysis via next-generation sequencing technology has brought new insight into the knowledge of whole transcriptomes in many organisms [23], [24], [25], [26], [27], [28]. This powerful approach allows the detection of significantly expressed genes and important pathways involved in different developmental stages and/or physiological processes [25], [27], [29], [30], [31], [32]. In addition, the large amount of transcriptomic sequences provides a rich resource for the discovery of different types of molecular markers [25], [26], [33], [34], [35], [36], [37].

Here, we conducted transcriptome analysis of Oujiang color common carp skin and obtained the first transcriptomic repertoire of this unique common carp strain. We also investigated the pigmentation genes expressed in the skin of Oujiang color varieties of common carp and found a set of candidate SNPs markers that can be used for selective molecular breeding in the future.

## Materials and Methods

### Ethics statement

This study was approved by the Institutional Animal Care and Use Committee (IACUS) of Shanghai Ocean University, Shanghai, China. The collection of fish samples was permitted by the Provincial Farm of Oujiang Color Common Carp in Zhejiang Province, China, and all sampling procedures were complied with the guidelines of IACUS on the care and use of animals for scientific purposes.

### Sample collection

Oujiang color common carp samples (tetraploid) were collected from six siblings, including three whole white (WW) and three red with big black spots (RB), from the Provincial Farm of Oujiang Color Common Carp in Zhejiang Province, China. Fresh skin tissues (∼3 g) were collected and preserved immediately in liquid nitrogen for RNA extraction.

### RNA extraction and mRNA purification

Each frozen sample was ground in mortars with liquid nitrogen, and total RNA was isolated using TRIzol reagent (Invitrogen) following the standard protocol. The concentration of total RNA dissolved in 200 µL RNase-free water was determined by NanoDrop (Thermo Scientific, USA), whereas the RNA integrity value (RIN) was estimated using RNA 6000 Pico LabChip of Agilent 2100 Bioanalyzer (Agilent, USA). Total RNA of each pooled sample of three individuals from the same color pattern was incubated with 10 U DNase I (Ambion) at 37°C for 1 hr, and then nuclease-free water was added to bring the sample volume to 250 µL. Messenger RNA was further purified with MicroPoly(A) Purist Kit (Ambion) as per the recommended protocol by the manufacturer. The mRNA was dissolved in 100 µL THE RNA Storage Solution, and the final concentration was determined using NanoDrop.

### cDNA synthesis

Double-stranded cDNA was synthesized from mRNA according to Ng's full-length cDNA synthesis protocol with minor modifications [38]. A GsuI-oligo dT primer was used to prime first-strand cDNA synthesis from 10 µg of mRNA, using 1000 units of Superscript II reverse transcriptase (Invitrogen). After incubation at 42°C for 1 hr, the 5′-CAP structure of mRNA was oxidized by NaIO4 (Sigma) and ligated to biotin hydrazide, which was used to select complete mRNA/cDNA by binding Dynal M280 beads (Invitrogen). After the second strand cDNA synthesis, the polyA and 5′ adaptor were removed through GsuI digestion.

### cDNA sequencing

cDNA size fractionation was performed using a cDNA size fractionation column (Agencourt). Each cDNA fraction larger than 800 bp was sonicated to the range of 300–800 bp, and then pooled together with the other cDNA samples within the same range of lengths. The prepared cDNA of each pooled sample of three individuals from the same color pattern was transformed into single-stranded template DNA (sstDNA) libraries by using the GS DNA Library Preparation Kit (Roche Applied Science). The sstDNA libraries were clonally amplified in a bead-immobilized form by using the GS emPCR Kit (Roche Applied Science) and sequenced on the Roche 454 Genome Sequencer FLX instrument.

### Sequence assembly and annotation

**Figure 1:**
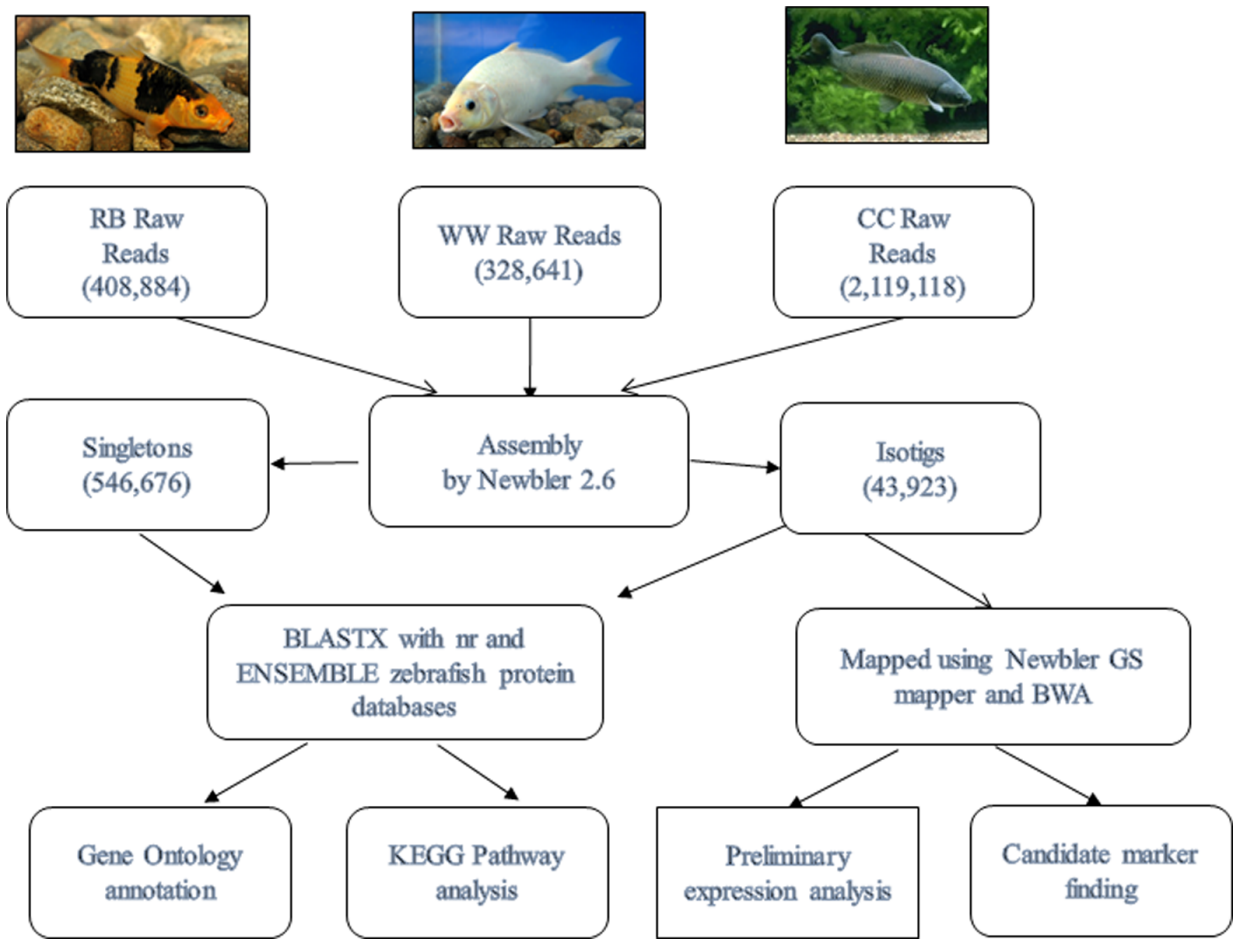
Analysis pipeline of Oujiang color varieties of common carp (RB - red with big black spots and WW – whole white) and gynogenic common carp (CC) transcriptomes, with the number of raw 454 reads, singletons and isotigs denoted. The pictures of RB, WW and CC are shown at the top panel. CC raw sequencing reads were downloaded from NCBI SRA database (SRA 050545).

Sequence assembly, downstream analysis and annotation were conducted following the pipeline illustrated in Figure 1. The raw sequencing reads generated in this study have been deposited to the NCBI Short Read Archive (SRA) database (SRA 291286). The reads from Oujiang color common carp (RB and WW) along with the gynogenic common carp (CC) originating from Roche 454 sequencing reads downloaded from NCBI (SRA050545) were assembled using Newbler 2.6 [39].

The assembled transcripts (isotigs and singletons) were BLASTed against NCBI nr protein database as well as zebrafish protein sequences, downloaded from ENSEMBL database, with a cutoff E-value smaller than 1e-10 [40]. Gene Ontology analysis was performed with the BLAST2GO program [41]. The BLAST program was also used to identify fish pigmentation genes [4], [6] in Oujiang color common carp transcriptome.

### Mapping and SNP Identification

All reads were mapped to the *de novo* common carp transcriptome assembled in this study to identify candidate SNP markers using Newbler GS mapper [39]. To find a consensus set of candidate SNPs markers, we also used BWA 0.6.1 [42] for mapping and FaSD [43] for SNPs detection. SNPs detected by the above two approaches and with at least three reads mapped were considered as putative markers specific to RB or WW.

## Results and Discussion

### Transcriptome of common carp and Oujiang color varieties

The statistics of common carp sequencing and assembly are summarized in Table 1. Altogether, 737,525 raw reads were generated via Roche 454 sequencing technology with an average length of 252 bp and maximal length of 680 bp. The reads generated in this study and the over two million Roche 454 sequencing reads from common carp available at NCBI SRA database (SRA050545) were assembled into 43,923 isotigs, with 546,676 reads unassembled (singletons). A total of 344,319 (63.0%) singletons were from the two color varieties of common carp and 75,260 singletons were found to have significant Blast hits. The N50 value, the average length and the largest length of isotigs were 1,237, 1,000 and 11,663 bp, respectively. The BLAST search resulted in 31,445 isotigs (71.6%) with significant matches to the NCBI nr database and 30,304 isotigs (68.9%) to ENSEMBL zebrafish proteins (E-value<1e-10). In addition, 12,597 zebrafish genes (about half of the zebrafish transcriptome) were found with significant BLAST matches to the assembled common carp transcriptome, indicating that approximately half of the common carp transcriptome can be annotated. For singletons, 128,507 out of 546,676 (23.5%) were found to have significant BLAST hits.

**Table 1:** Summary statistics of common carp skin transcriptomic sequencing and assembly.

Raw Reads	
RB*	408,884
WW*	328,641
CC**	2,119,118
Total	2,856,643
Number of isotigs	43,923
Number of singletons	546,676
Average contig length (bp)	1,000
Maximal contig length (bp)	11,663

* Oujiang color varieties of common carp: RB - red with big black spots, WW - whole white;

** CC reads are from a gynogenic common carp from 12 tissues downloaded from NCBI (SRA 050545).

**Figure 2:**
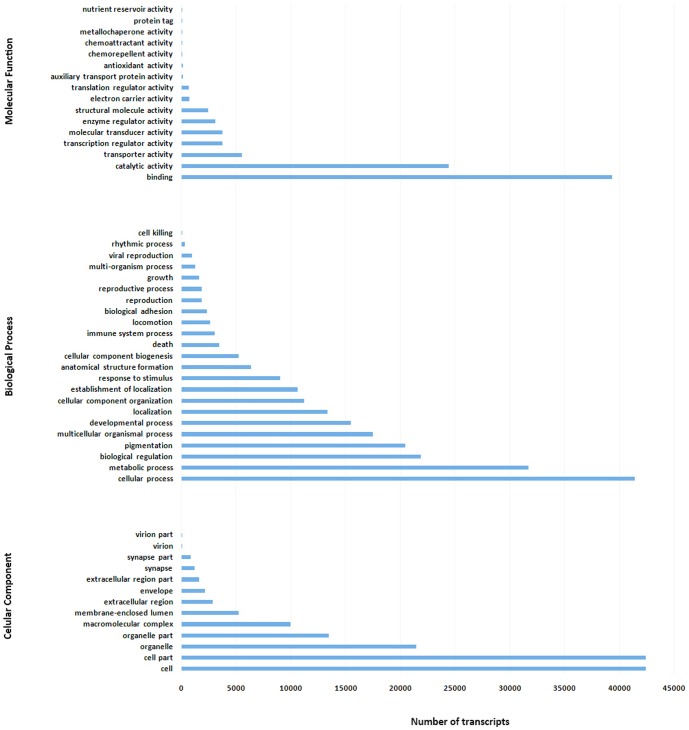
The number of common carp transcripts in each level-2 Gene Ontology category. The transcriptome was assembled from Oujiang color varieties of common carp (RB - red with big black spots and WW – whole white) and gynogenic common carp (CC).

Further analysis of Gene Ontology (GO) showed that 25,564 isotigs and 45,383 singletons were successfully mapped to Gene Ontology categories - molecular functions, biological processes, and cellular components (Figure 2). For molecular functions, the majority of the transcripts (including both isotigs and singletons) were associated with binding, protein binding, and catalytic activity. For cellular component, the final products of these transcripts (proteins) were located mainly within cell, which is probably not surprising because the skin tissue was sampled for RNA extraction. For biological processes, most of these transcripts were related to cellular process and metabolic process, which was in agreement with most of other studies [24], [44]. A total of 134 KEGG pathways were found in the common carp transcriptome with the top 20 pathways shown in Table 2. The pathways with most genes represented were purine metabolism, glycolysis and gluconeogenesis, and oxidative phosphorylation. Tyrosine metabolism, a pigmentation related pathway, was found with 37 isotigs and 43 singletons that correspond to 23 proteins.

**Table 2:** The number of common carp transcripts (isotigs and singletons) and enzymes found in each KEGG pathway.

Pathways	No. of isotigs	No. of enzymes (isotigs)	No. of singletons	No. of enzymes (singletons)
Purine metabolism	477	55	1,263	55
Glycolysis/Gluconeogenesis	196	16	177	24
Oxidative phosphorylation	175	8	445	7
Pyrimidine metabolism	166	30	387	28
Amino sugar and nucleotide sugar metabolism	147	37	94	22
Fructose and mannose metabolism	122	19	78	12
Phosphatidylinositol signaling system	121	18	284	17
Arginine and proline metabolism	121	28	151	28
Aminoacyl-tRNA biosynthesis	119	21	96	23
Pyruvate metabolism	110	24	110	15
Pentose phosphate pathway	108	17	81	14
Carbon fixation pathways in prokaryotes	106	20	119	16
Starch and sucrose metabolism	98	22	93	21
Methane metabolism	96	19	87	16
Inositol phosphate metabolism	94	20	193	17
Glycerophospholipid metabolism	93	26	171	21
T cell receptor signaling pathway	93	2	167	2
Lysine degradation	93	15	152	14
Citrate cycle (TCA cycle)	92	17	100	16
Cysteine and methionine metabolism	89	25	90	19

Shown only the top 20 pathways and see Table S2 for the complete list of 134 pathways.

In our study, approximately 30% of the isotigs and 80% of the singletons were found without significant BLAST matches to known proteins in public databases. In fact, most transcriptome studies showed a portion of isotig and singleton sequences that cannot be annotated [24], [25], [26], [34], [35], [45]. One possible reason for this is that these non-model organisms possess numerous putative novel genes or transcripts, of which the sequences are not available in public databases. Further analysis and annotation of these putative novel transcripts are necessary, but appear to be challenging. With the availability of transcriptomic sequences in many organisms, the comparative analysis of closely related species may assist in the identification of novel orthologous genes. Besides the widely used BLAST algorithm, the profile- or model-based approaches such as PRINTS [46], PROSITE [47], and Pfam [48] can be used to predict the functions of isotig/singleton sequences.

### Pigmentation genes in Oujiang color varieties of common carp

After a BLAST search with 145 zebrafish-derived pigmentation genes against the common carp transcriptome, 593 isotigs were found with hits to 99 pigmentation genes and 501 singletons with matches to 95 pigmentation genes (E-value<1e-10). Of the 501 singletons, 291 were from WW and 210 from RB. A total of 77 pigmentation genes were commonly found in both isotigs and singletons, with 22 occurring solely in isotigs and 18 found only in singletons. When comparing the mapped reads of these pigmentation genes in RB and WW, 187 isotigs were found with at least a two-fold change, including 106 higher in WW and 81 in RB. In addition, 134 RB isotigs were not found in WW whereas 100 WW isotigs were not found in RB (Table S1).

### Isolation of SNP markers specific to the RB or WW color varieties

**Figure 3:**
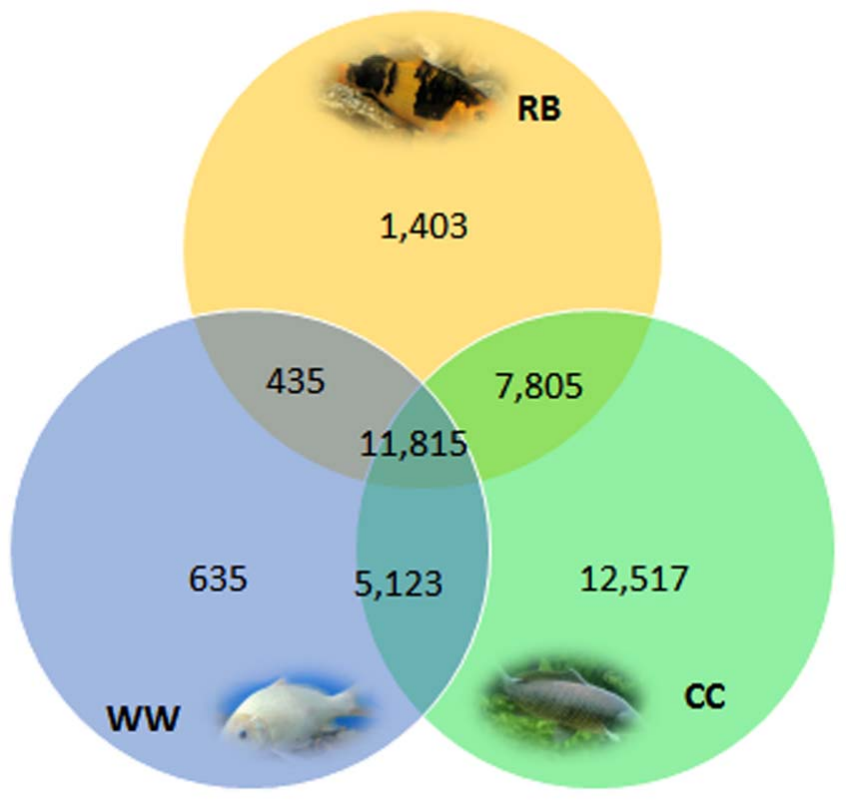
Venn diagram of the number of isotigs found in the skin transcriptome of Oujiang color varieties of common carp (RB and WW) and gynogenic transcriptome of common carp (CC). RB - red with big black spots, WW - whole white.

Reads from Oujiang color varieties of common carp and from NCBI SRA database were mapped to the assembled *de novo* common carp transcriptome. Over 11,000 isotigs were mapped with reads from RB, WW, and CC (Figure 3). There were about 1,403 and 635 isotigs uniquely mapped with RB and WW reads, respectively, suggesting that more specific genes were involved in the formation of RB phenotype compared with WW. Many more unique isotigs were mapped with reads only from CC, which may be due to the much larger number of original reads from CC (Figures 1 and 3).

**Figure 4:**
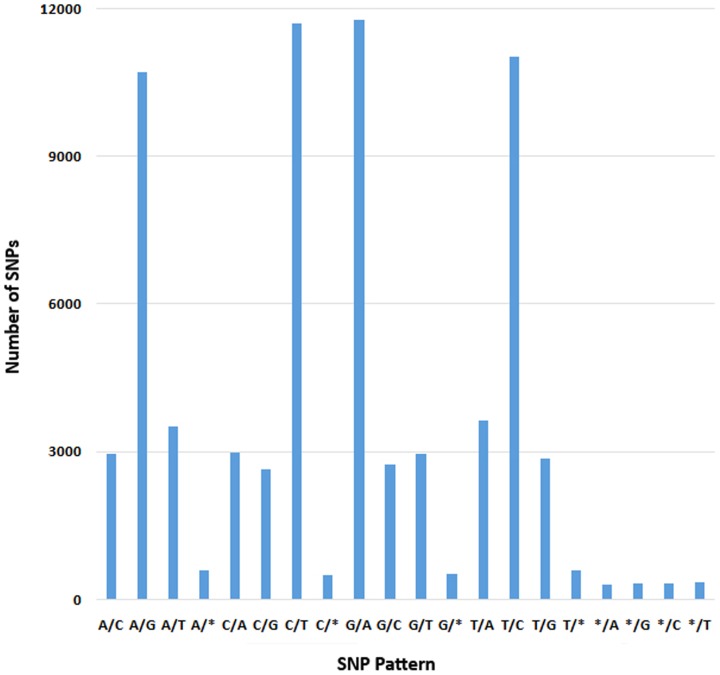
The distribution of SNP types in the common carp transcriptome. * indicates single nucleotide polymorphisms other than single nucleotide substitution, including deletion.

A total of 52,902 single nucleotide polymorphism (SNP) markers were found in 9,379 isotigs, including 3,721 single nucleotide insertions or deletions (indels). The frequency distribution of different substitutions or indels is shown in Figure 4. The highest SNP pattern was G/A, accounting for 16% of the total. Compared to other substitution types, the substitution between C and G was found to be the least frequent.

To discover reliable markers that can be used to distinguish RB and WW, we set up the following three criteria: 1) detected with two algorithms; 2) homozygous in either RB or WW; and 3) more than two mapped reads. When these criteria were applied, 63 putative SNPs markers were found, including 21 SNPs with RB showing the reference nucleotide, while WW showing the variant one and 42 SNPs with the opposite (Table 3). In addition, the analysis of pigmentation-related transcripts revealed only 10 SNPs that may be associated with different coloration phenotypes. One of our future research directions is to validate the predicted markers and evaluate their accuracy in identifying specific Oujiang color varieties of common carp with distinct color patterns [36], [37], [49].

**Table 3:** Putative color pattern-specific SNP markers found in Oujiang color varieties of common carp isotig sequences generated from the skin transcriptome*.

No. Isotigs	Location	Reference/Variant	RB**	WW**
Isotig11776	721	C/T	11/0	0/3
Isotig21770	1093	A/T	10/0	0/9
Isotig20802	2,791	G/A	10/0	0/5
Isotig11776	1,203	A/G	9/0	0/3
Isotig23531	771	C/G	8/0	0/5
Isotig12829	341	A/G	7/0	0/3
Isotig23531	483	C/T	7/0	0/3
Isotig21113	1,496	A/G	6/0	0/4
Isotig20807	2,081	A/G	5/0	0/5
Isotig21311	779	C/T	4/0	0/7

* See Table S3 for the complete list of candidate SNP markers.

** RB - red with big black spots, WW - whole white.

## Conclusions

The *de novo* skin transcriptome sequencing of Oujiang color common carp varieties were conducted, resulting in 737,525 reads assembled to 43,923 isotigs. Over 10,000 genes were annotated using BLAST, Gene Ontology, and KEGG pathways. The orthologs of approximately 80% (117 out of 145) zebrafish pigmentation genes were recovered. In addition, 52,902 SNPs were detected, with 63 candidate markers predicted. Future research directions include expanding this study to analyze more samples of other color patterns and validating the results on potentially differentially expressed genes between the RB and WW phenotypes. We are also planning to validate color pattern-specific molecular markers, which will presumably lead to a better understanding of the molecular mechanisms underlying diverse color patterns and promote aquaculture of Oujiang color varieties of common carp through the application of molecular markers in selective breeding.

## Supporting Information

Table S1 **Pigmentation genes found in Oujiang color varieties of common carp (RB - red with big black spots and WW - whole white).**
(XLSX)

Table S2 **The complete list of KEGG pathways identified in the common carp skin transcriptome involving** two Oujiang color common carp variants.
(DOCX)

Table S3 **The complete list of putative SNP markers found in Oujiang color varieties of common carp (RB - red with big black spots, WW - whole white).**
(DOCX)
