# Supplementary material for: Analysis of the Skin Transcriptome in Two Oujiang Color Varieties of Common Carp
Source: PLoS One. 2014 Mar 6;9(3):e90074. doi: 10.1371/journal.pone.0090074 (PMC3946065; doi:10.1371/journal.pone.0090074)
Supplement: Table S2 — The complete list of KEGG pathways identified in the common carp skin transcriptome involving two Oujiang color common carp variants. (DOCX) [file pone.0090074.s002.docx]

Table S2. Complete list of **KEGG pathway identified in common carp transcriptome**

| Pathways | No. Sequences(Isotig) | No. enzymes(Isotig) | No. Sequences(Singletons) | No. enzymes(Singletons) |
| --- | --- | --- | --- | --- |
| Purine metabolism | **477** | **55** | **1263** | **55** |
| Glycolysis / Gluconeogenesis | **196** | **16** | **177** | **24** |
| Oxidative phosphorylation | **175** | **8** | **445** | **7** |
| Pyrimidine metabolism | **166** | **30** | **387** | **28** |
| Amino sugar and nucleotide sugar metabolism | **147** | **37** | **94** | **22** |
| Fructose and mannose metabolism | **122** | **19** | **78** | **12** |
| Phosphatidylinositol signaling system | **121** | **18** | **284** | **17** |
| Arginine and proline metabolism | **121** | **28** | **151** | **28** |
| Aminoacyl-tRNA biosynthesis | **119** | **21** | **96** | **23** |
| Pyruvate metabolism | **110** | **24** | **110** | **15** |
| Pentose phosphate pathway | **108** | **17** | **81** | **14** |
| Carbon fixation pathways in prokaryotes | **106** | **20** | **119** | **16** |
| Starch and sucrose metabolism | **98** | **22** | **93** | **21** |
| Methane metabolism | **96** | **19** | **87** | **16** |
| Inositol phosphate metabolism | **94** | **20** | **193** | **17** |
| Glycerophospholipid metabolism | **93** | **26** | **171** | **21** |
| T cell receptor signaling pathway | **93** | **2** | **167** | **2** |
| Lysine degradation | **93** | **15** | **152** | **14** |
| Citrate cycle (TCA cycle) | **92** | **17** | **100** | **16** |
| Cysteine and methionine metabolism | **89** | **25** | **90** | **19** |
| Glycine, serine and threonine metabolism | **87** | **29** | **92** | **20** |
| Propanoate metabolism | **87** | **16** | **61** | **12** |
| Phenylalanine metabolism | **85** | **9** | **48** | **13** |
| Galactose metabolism | **84** | **21** | **77** | **13** |
| Alanine, aspartate and glutamate metabolism | **81** | **22** | **97** | **24** |
| Arachidonic acid metabolism | **81** | **12** | **61** | **12** |
| Glutathione metabolism | **77** | **15** | **66** | **14** |
| Carbon fixation in photosynthetic organisms | **76** | **13** | **54** | **9** |
| Valine, leucine and isoleucine degradation | **75** | **17** | **63** | **15** |
| Butanoate metabolism | **68** | **18** | **51** | **3** |
| Glycerolipid metabolism | **67** | **18** | **109** | **15** |
| Tryptophan metabolism | **63** | **12** | **98** | **17** |
| Thiamine metabolism | **61** | **4** | **163** | **2** |
| Fatty acid degradation | **61** | **12** | **77** | **13** |
| Drug metabolism - other enzymes | **59** | **12** | **129** | **15** |
| Glyoxylate and dicarboxylate metabolism | **57** | **14** | **70** | **15** |
| Biosynthesis of unsaturated fatty acids | **50** | **8** | **23** | **8** |
| Sphingolipid metabolism | **49** | **13** | **95** | **16** |
| Nicotinate and nicotinamide metabolism | **49** | **12** | **57** | **11** |
| beta-Alanine metabolism | **48** | **11** | **47** | **10** |
| Aminobenzoate degradation | **45** | **8** | **61** | **6** |
| One carbon pool by folate | **40** | **17** | **75** | **13** |
| Other glycan degradation | **40** | **8** | **40** | **5** |
| alpha-Linolenic acid metabolism | **40** | **5** | **37** | **5** |
| Fatty acid elongation | **40** | **8** | **32** | **8** |
| Ether lipid metabolism | **38** | **10** | **54** | **6** |
| Tyrosine metabolism | **37** | **10** | **43** | **13** |
| Phenylpropanoid biosynthesis | **37** | **1** | **9** | **2** |
| Drug metabolism - cytochrome P450 | **36** | **6** | **39** | **6** |
| Various types of N-glycan biosynthesis | **34** | **7** | **69** | **10** |
| mTOR signaling pathway | **34** | **3** | **51** | **12** |
| Metabolism of xenobiotics by cytochrome P450 | **34** | **5** | **38** | **6** |
| Geraniol degradation | **34** | **4** | **25** | **3** |
| Caprolactam degradation | **34** | **4** | **21** | **2** |
| N-Glycan biosynthesis | **33** | **10** | **87** | **11** |
| Benzoate degradation | **33** | **4** | **13** | **3** |
| Fatty acid biosynthesis | **32** | **9** | **40** | **7** |
| Streptomycin biosynthesis | **31** | **7** | **25** | **4** |
| Ascorbate and aldarate metabolism | **31** | **5** | **11** | **4** |
| Porphyrin and chlorophyll metabolism | **30** | **14** | **47** | **12** |
| Primary bile acid biosynthesis | **29** | **2** | **25** | **4** |
| Toluene degradation | **29** | **2** | **22** | **2** |
| Steroid hormone biosynthesis | **25** | **9** | **57** | **12** |
| Other types of O-glycan biosynthesis | **23** | **8** | **46** | **7** |
| Terpenoid backbone biosynthesis | **23** | **10** | **37** | **11** |
| Nitrogen metabolism | **22** | **4** | **22** | **5** |
| Limonene and pinene degradation | **22** | **1** | **11** | **2** |
| Retinol metabolism | **21** | **9** | **32** | **10** |
| Glycosphingolipid biosynthesis - ganglio series | **20** | **6** | **32** | **5** |
| Synthesis and degradation of ketone bodies | **20** | **5** | **11** | **3** |
| Peptidoglycan biosynthesis | **20** | **10** | **1** | **1** |
| Selenocompound metabolism | **18** | **6** | **45** | **6** |
| Glycosaminoglycan biosynthesis - heparan sulfate / heparin | **18** | **5** | **42** | **9** |
| Glycosphingolipid biosynthesis - globo series | **18** | **5** | **18** | **4** |
| Pentose and glucuronate interconversions | **18** | **12** | **18** | **7** |
| Pantothenate and CoA biosynthesis | **17** | **7** | **43** | **9** |
| C5-Branched dibasic acid metabolism | **17** | **1** | **11** | **2** |
| Phenylalanine, tyrosine and tryptophan biosynthesis | **17** | **8** | **8** | **4** |
| Butirosin and neomycin biosynthesis | **17** | **1** | **5** | **1** |
| Linoleic acid metabolism | **15** | **3** | **35** | **3** |
| Glycosaminoglycan biosynthesis - chondroitin sulfate / dermatan sulfate | **15** | **7** | **26** | **8** |
| Isoquinoline alkaloid biosynthesis | **15** | **3** | **22** | **7** |
| Tropane, piperidine and pyridine alkaloid biosynthesis | **15** | **3** | **16** | **3** |
| Riboflavin metabolism | **14** | **2** | **40** | **5** |
| Cyanoamino acid metabolism | **14** | **6** | **19** | **4** |
| Glycosaminoglycan biosynthesis - keratan sulfate | **13** | **5** | **38** | **5** |
| Mucin type O-Glycan biosynthesis | **13** | **4** | **33** | **6** |
| Glycosphingolipid biosynthesis - lacto and neolacto series | **12** | **5** | **24** | **8** |
| Steroid biosynthesis | **11** | **7** | **26** | **8** |
| Taurine and hypotaurine metabolism | **10** | **6** | **18** | **4** |
| Sulfur metabolism | **10** | **7** | **13** | **6** |
| Biotin metabolism | **10** | **5** | **9** | **3** |
| Aflatoxin biosynthesis | **9** | **1** | **22** | **1** |
| Tetracycline biosynthesis | **9** | **1** | **22** | **1** |
| Caffeine metabolism | **9** | **4** | **21** | **4** |
| D-Glutamine and D-glutamate metabolism | **9** | **5** | **13** | **2** |
| Novobiocin biosynthesis | **9** | **2** | **6** | **2** |
| Folate biosynthesis | **8** | **7** | **23** | **5** |
| Chloroalkane and chloroalkene degradation | **8** | **2** | **10** | **2** |
| Ubiquinone and other terpenoid-quinone biosynthesis | **8** | **4** | **7** | **4** |
| Naphthalene degradation | **8** | **1** | **5** | **1** |
| Histidine metabolism | **7** | **3** | **29** | **8** |
| Cutin, suberine and wax biosynthesis | **6** | **2** | **7** | **2** |
| Vitamin B6 metabolism | **6** | **4** | **5** | **2** |
| Polyketide sugar unit biosynthesis | **6** | **2** | **3** | **1** |
| Valine, leucine and isoleucine biosynthesis | **5** | **2** | **6** | **2** |
| Lysine biosynthesis | **5** | **5** | **0** | **0** |
| Biosynthesis of ansamycins | **4** | **1** | **4** | **1** |
| Flavone and flavonol biosynthesis | **4** | **1** | **2** | **1** |
| Phosphonate and phosphinate metabolism | **4** | **3** | **1** | **1** |
| Styrene degradation | **4** | **3** | **1** | **1** |
| Biosynthesis of vancomycin group antibiotics | **4** | **1** | **0** | **0** |
| Ethylbenzene degradation | **3** | **1** | **5** | **1** |
| Glucosinolate biosynthesis | **3** | **1** | **5** | **1** |
| Glycosylphosphatidylinositol(GPI)-anchor biosynthesis | **3** | **1** | **5** | **1** |
| Indole alkaloid biosynthesis | **3** | **1** | **4** | **1** |
| D-Alanine metabolism | **3** | **2** | **0** | **0** |
| Lipopolysaccharide biosynthesis | **3** | **2** | **0** | **0** |
| Steroid degradation | **2** | **1** | **18** | **3** |
| PI3K-Akt signaling pathway | **2** | **1** | **10** | **1** |
| Bisphenol degradation | **2** | **1** | **3** | **1** |
| Lipoic acid metabolism | **2** | **1** | **3** | **1** |
| Penicillin and cephalosporin biosynthesis | **2** | **1** | **2** | **1** |
| beta-Lactam resistance | **2** | **1** | **0** | **0** |
| Dioxin degradation | **2** | **1** | **0** | **0** |
| Photosynthesis | **2** | **1** | **0** | **0** |
| Xylene degradation | **2** | **1** | **0** | **0** |
| Nitrotoluene degradation | **1** | **1** | **4** | **2** |
| Betalain biosynthesis | **1** | **1** | **3** | **2** |
| Sesquiterpenoid and triterpenoid biosynthesis | **1** | **1** | **3** | **1** |
| Biosynthesis of siderophore group nonribosomal peptides | **1** | **1** | **0** | **0** |
| Glycosaminoglycan degradation | **0** | **0** | **50** | **7** |
| D-Arginine and D-ornithine metabolism | **0** | **0** | **2** | **1** |
| Melanogenesis | **0** | **0** | **1** | **1** |
