# Supplementary material for: Analysis of the Skin Transcriptome in Two Oujiang Color Varieties of Common Carp
Source: PLoS One. 2014 Mar 6;9(3):e90074. doi: 10.1371/journal.pone.0090074 (PMC3946065; doi:10.1371/journal.pone.0090074)
Supplement: Table S3 — The complete list of putative SNP markers found in Oujiang color varieties of common carp (RB - red with big black spots, WW - whole white). (DOCX) [file pone.0090074.s003.docx]

**Table S3** Complete list of putative SNP markers found in Oujiang color common carp (RB - red with big black spots, WW - whole white)

| **Isotig#** | **Location** | **Base/Substitution** | **RB** | **WW** |
| --- | --- | --- | --- | --- |
| **Isotig11776** | 721 | C/T | 11/0 | 0/3 |
| **Isotig21770** | 1093 | A/T | 10/0 | 0/9 |
| **Isotig20802** | 2791 | G/A | 10/0 | 0/5 |
| **Isotig11776** | 1203 | A/G | 9/0 | 0/3 |
| **Isotig23531** | 771 | C/G | 8/0 | 0/5 |
| **Isotig12829** | 341 | A/G | 7/0 | 0/3 |
| **Isotig23531** | 483 | C/T | 7/0 | 0/3 |
| **Isotig21113** | 1496 | A/G | 6/0 | 0/4 |
| **Isotig20807** | 2081 | A/G | 5/0 | 0/5 |
| **Isotig21311** | 779 | C/T | 4/0 | 0/7 |
| **Isotig12372** | 1175 | A/G | 4/0 | 0/4 |
| **Isotig11987** | 768 | G/A | 4/0 | 0/4 |
| **Isotig22586** | 1017 | C/A | 3/0 | 0/4 |
| **Isotig24389** | 679 | T/C | 3/0 | 0/4 |
| **Isotig24389** | 688 | T/A | 3/0 | 0/4 |
| **Isotig06897** | 969 | A/G | 3/0 | 0/3 |
| **Isotig24389** | 655 | A/G | 3/0 | 0/3 |
| **Isotig24389** | 661 | A/G | 3/0 | 0/3 |
| **Isotig12016** | 2214 | C/T | 3/0 | 0/3 |
| **Isotig24389** | 667 | C/A | 3/0 | 0/3 |
| **Isotig05677** | 1344 | T/C | 3/0 | 0/3 |
| **Isotig05756** | 692 | C/A | 0/8 | 3/0 |
| **Isotig21762** | 806 | T/C | 0/7 | 18/0 |
| **Isotig26017** | 173 | T/A | 0/7 | 7/0 |
| **Isotig05756** | 574 | A/C | 0/7 | 3/0 |
| **Isotig26062** | 712 | C/T | 0/6 | 3/0 |
| **Isotig23199** | 1025 | C/T | 0/5 | 8/0 |
| **Isotig20807** | 2063 | G/T | 0/5 | 5/0 |
| **Isotig10551** | 1344 | G/A | 0/5 | 4/0 |
| **Isotig25339** | 465 | G/C | 0/5 | 4/0 |
| **Isotig36609** | 153 | C/T | 0/5 | 3/0 |
| **Isotig25053** | 481 | T/C | 0/5 | 3/0 |
| **Isotig21762** | 977 | A/G | 0/4 | 12/0 |
| **Isotig20802** | 2608 | C/T | 0/4 | 5/0 |
| **Isotig20802** | 2620 | G/A | 0/4 | 5/0 |
| **Isotig08695** | 1582 | C/T | 0/4 | 4/0 |
| **Isotig13940** | 296 | C/T | 0/4 | 4/0 |
| **Isotig21851** | 681 | C/T | 0/4 | 4/0 |
| **Isotig21487** | 304 | A/G | 0/4 | 3/0 |
| **Isotig13940** | 281 | C/T | 0/4 | 3/0 |
| **Isotig21975** | 1044 | G/A | 0/4 | 3/0 |
| **Isotig24186** | 340 | G/A | 0/4 | 3/0 |
| **Isotig42205** | 136 | T/G | 0/4 | 3/0 |
| **Isotig20946** | 721 | A/G | 0/3 | 9/0 |
| **Isotig21762** | 260 | C/T | 0/3 | 9/0 |
| **Isotig21762** | 246 | G/T | 0/3 | 9/0 |
| **Isotig21119** | 69 | G/T | 0/3 | 5/0 |
| **Isotig11637** | 2112 | C/T | 0/3 | 4/0 |
| **Isotig23627** | 138 | C/T | 0/3 | 4/0 |
| **Isotig21669** | 1282 | T/C | 0/3 | 4/0 |
| **Isotig09488** | 382 | A/T | 0/3 | 3/0 |
| **Isotig21487** | 517 | A/G | 0/3 | 3/0 |
| **Isotig28921** | 423 | A/G | 0/3 | 3/0 |
| **Isotig33368** | 290 | A/G | 0/3 | 3/0 |
| **Isotig22156** | 1097 | C/T | 0/3 | 3/0 |
| **Isotig23544** | 698 | C/T | 0/3 | 3/0 |
| **Isotig26805** | 208 | C/T | 0/3 | 3/0 |
| **Isotig20802** | 3526 | G/A | 0/3 | 3/0 |
| **Isotig23627** | 192 | G/T | 0/3 | 3/0 |
| **Isotig23627** | 198 | G/A | 0/3 | 3/0 |
| **Isotig33368** | 298 | G/A | 0/3 | 3/0 |
| **Isotig28921** | 439 | T/C | 0/3 | 3/0 |
| **Isotig28921** | 441 | T/C | 0/3 | 3/0 |
